# Some Vital Phases of Fractures of the Jaws*Read before the Section on Stomatology at the Sixty-Ninth Annual Session of the American Medical Association, Chicago, June, 1918. Reprinted from *Journal A. M. A.* for July 20, 1918.

**Published:** 1918-08

**Authors:** Chalmers J. Lyons


### SOME VITAL PHASES OF FRACTURES OF THE JAWS *

CHALMERS J. LYONS, D.D.SC.

The subject of fractures of the jaws is one that has attracted the attention of medicine since the days of Hippocrates. It also is a subject that was of considerable interest to dentistry long before it became a profession. Indeed, many of the principles laid down by Hippocrates can be used today with a moderate degree of success. It was Hippocrates who first originated interdental ligation. It was also Hippocrates who condemned the use of bandages alone as a procedure in the treatment of fractures of the jaws.

In presenting the subject of fractures of the jaws at this time, we shall touch on only two or three vital phases of the subject. The first phase which we shall attempt to discuss will be the process of repair. We use the word "discuss" advisedly, because in our present state of knowledge of the subject, we can only discuss it. We are not in a position to make any dogmatic statements as to just what takes place in the process of repair of bone.

Just what takes place in the behavior of the tissues in the process of repair of bone is a question offering a diversity of opinion. There seems to be an antagonistic state of opinion about the theory of bone growth. There are two schools that are contending for the maintenance of their theories along this line: One contends that the periosteum is a limiting membrane without the property of osteogenesis; the other maintains that the periosteum is an osteogenetic membrane and can go on functionating, developing and nourishing new bone.

---

* Read before the Section on Stomatology at the Sixty-Ninth Annual Session of the American Medical Association, Chicago, June, 1918. Reprinted from *Journal A. M. A.* for July 20, 1918.

Havers, in 1692, gave the first accurate account of osseous structure and described the periosteum as simply a connective tissue, limiting and vascularizing membrane.

Antoine de Heyde, in 1684, made some observations on frogs and determined that callus was formed by calcification of a blood clot extravasated around broken bone ends.

In the middle of the eighteenth century, Duharnel brought out the generally accepted theory of the function of the periosteum. His view was that the periosteum became thickened and succulent around a fracture and by pushing the new tissue in among the fragments it formed a callus. In his experiments, he discovered a layer of cells lying next to the bone. To this layer, he gave the name cambium layer. This layer of cells between the true periosteum and the bone is recognized in the bone work now being done in Europe.

MacEwen is emphatic in the statements that the function of the periosteum is simply a limiting membrane and does not have the property of osteogenesis. He made many experiments on dogs. In these he seems to prove that the periosteum does not produce new bone. In many instances, he reports that the whole radius was removed, leaving the periosteum. He finds in these cases that after several weeks no new bone is formed. In his experiments, he finds no place where the periosteal flaps produced new bone. He also finds that where the periosteum was not intact, there was a marked hypertrophy of the bone, showing that periosteum limits bone growth.

These experiments of MacEwen are substantiated by similar experiments by Cohen and Mann. Their experiments included transplanting of bone denuded of periosteum into muscle, into the medullary canal and into newly made bone defects. Some of these transplants were covered with periosteum and others were not. It was found that isolated bone grafts did not act as foreign bodies and were not absorbed after sixty days, but showed a tendency to outgrowth. They also made experiments in which the periosteum per se was transplanted around the carotid artery. This did not show bone proliferation. They came to the conclusion that the periosteum was not at all essential to the healing of a fracture.

Other investigators in this country have made similar experiments with similar results. These investigations, with others, represent the work of the contenders of the theory that the periosteum is simply a limiting membrane. The other class of investigators in bone work contend that these experiments must have been carried out with simply the outer layer of the periosteum, which is a limiting membrane, and that the inner layer, or the cambium layer, the layer which Hey Groves terms the epiostial layer, or the osteogenetic layer of the periosteum, was not considered.

Our experiments at the University of Michigan hospitals do not accord with the theory of MacEwen. We have found that periosteum transplanted into the muscle tissue will produce new bone. This new growth seems to have all the properties of true bone; its blood supply, its bone cells and the process of true osteogenesis seem to be normal.

This deposition of bone differs from other calcific deposits which may take place in the body, such as in the spleen, liver, kidney, etc., under certain pathologic conditions, so that we feel quite positive that under suitable conditions, true bone may form from the periosteum.

Hey Groves summarizes as follows:

"The periosteum is chiefly a limiting membrane of the bone. The dense bone can live, grow, undergo repair, and produce fresh periosteum after the latter has been removed. In young bones it is possible to remove the periosteum in such a way as to produce an osteogenetic membrane, this being probably due to the lifting up of the epiosteum with the periosteum. In adult bones this is impossible except after trauma or an inflammation."

In the repair of fractures of the jaws, we feel that the retention of the periosteum is highly desirable, because its removal takes away much of the epiosteum, and, also, because it affords a ready means of vascularization.

While there is a wide diversity of opinion regarding the function of the periosteum in bone repair, all investigators seem to agree as to the function of the compact and cancellous bone in the process of repair.

Ollier proved that quite apart from the periosteum and the marrow, compact bone could live and produce new bone and undergo the callus repair of fracture. The deep surfaces of bone, like the superficial surfaces, are capable of osteogenesis under suitable stimulus.

Axhausen has shown that the wide haversian canals contain active osteoblasts and favorable conditions for new bone formation. It has been observed in gunshot fractures in France that when the ends of the fragments have been exposed and laid bare, by stimulating the ends with a drill, in a short time buttons of granulation tissue would be pushed out and would soon cover the entire ends of the bones. These ends could then be put in apposition, and union of fragments would result. In our clinics, we have had to resort to this method in some old ununited fractures, in which we have had the process of repair again started. It is our opinion that compact bone, if it has the proper blood supply, is quite independent of either endosteum or periosteum for bone growth and for bone repair.

The whole process of repair of bone is fundamentally that which takes place in the union of the soft parts. In fractures of the bone we may have primary union, or, we may have secondary union by granulation tissue; this granulation tissue differs from granulation tissue of the soft parts in that it is osseous in character. The injured tissues, infiltrated with blood, soon become invaded by leukocytes and effused blood plasma. Firbrinous coagulation takes place and the ends of the fragments are embedded in a dense, ill-defined mass of firm, cellular exudate. The periosteum becomes much thicker, softer and more vascular; a thin layer of gelatinous or viscid liquid is found between it and the bone for a short distance from the edge of the fracture. In about fourteen days, the effused blood is completely absorbed, leaving a firm, dense, cellular, vascularized, partly organized mass of granulation tissue. The bone then undergoes rarefying osteitis, and the fracture becomes fixed. This is known as the "provisional callus."

While this process is going on, similar changes are taking place in the cancellous bone, and the "internal callus" is formed in the same manner. Ossification then takes place, thus completing the process of repair. While the callus is forming, the process of repair is going on in the contiguous soft parts, and they regain their normal condition and function.

Very briefly, the foregoing is what seems to take place in the process of repair of fracture of the jaws. Occasionally there may be an excess of rarefying osteitis, and a lack of production of osteoblasts, so that the callus may not ossify. In these cases, the bone is absorbed for a considerable distance between the ends of the fragments, and we have established a false joint or pseudo-arthrosis.

When an open or compound fracture becomes infected, suppuration ensues, and the process of repair is slower, because the suppuration of the wound delays or prevents the formation of the provisional callus, and it has to depend on the formation of the internal callus, which is not so favorable for rapid repair. In these cases, the callus is larger and more irregular than that which we see after simple fractures, when the process of repair takes its normal course.

Another phase of this subject which we shall consider briefly is some of the complications which occur in fracture of the jaws. Fractures of the jaws will differ from fractures in other parts of the body in that they are more liable to infection on account of the close proximity to the bacteria-laden fluids of the oral cavity. We rarely find infection present in simple fractures of the jaws, but it is quite common in compound fractures.

A factor which must be considered in relation to infection in fractures of the jaws is the presence of alveolar abscesses, which may be existing at the time of the fracture or may be superinduced by the injury. These will greatly delay the process of repair and should be eradicated before repair can be expected to take place.

In fracture of the maxilla, the antrum is frequently involved and may become infected. This makes another serious complication. It means clearing up the infection in the antrum before the process of repair will go on in a normal way.

Another rare complication in fracture of the maxilla is the fracture of the brain case, in which coma or even death may result immediately. Secondary hemorrhage is one of the complicating problems that is quite common in fractures of the jaws on the battle fields of Europe. This comes on after suppuration has been established and is the result of infective inflammation causing a disintegration of the hemostatic thrombosis, or ulceration or sloughing of the walls of the vessels. This secondary hemorrhage may occur any time between the beginning of the process of repair and the complete repair of the fractured blood vessels.

The laceration or severing of sensitive nerve trunks will lead to anesthesia of all of the parts peripheral to the fracture, and neuralgia is a common sequela.

Trismus is an early local complication that is nearly always present. This is usually brought on by the violence that is produced on the soft tissues and the temporo-mandibular articulation. In extreme cases, where it is not possible to make the reduction at once, the trismus may be so pronounced that a general anesthetic may have to be resorted to in order to obtain sufficient relaxation to make a diagnosis and subsequent reduction.

In gunshot fractures, there are other local complications, such as extensive laceration of the soft parts, which will greatly add to the difficulty of making a diagnosis and prosecuting the treatment. In these cases, there will usually be greater splintering and fissuring of the bone than in the fractures in civil practice. It is reported that in every case of gunshot fracture of the jaws, infection of the wound ensues immediately, which greatly interferes with the process of repair.

In the work at the front on fracture of the jaws, the history shows that many of these cases are not treated until some time after the injury. Here a new complication arises. In these cases, frequently large masses of cicatricial tissue have formed, and a marked deformity is present. Under these conditions, a clear and definite diagnosis may be difficult, and the successful reduction of the fracture made arduous.

Another local complication, which is almost universal in gunshot fractures of the jaw and sometimes met with in civil practice, is loss of substance of the bone. Here again difficulties arise in making a successful reduction and subsequently retaining the fragments in normal relation.

Under the general complications of fractures of the jaws, there are a few factors that must not be overlooked. There are certain diseases which, when present, seem to hinder the process of repair. Such diseases as syphilis, alcoholism, tuberculosis and such chronic diseases as will cause a marked lowering of the vitality of the patient. These conditions will always delay union and may prevent it entirely.

It will not be out of place, perhaps, in this brief paper to say something regarding the prognosis in fractures of the jaw. The prognosis must vary greatly according to the location of the fracture, the character of it, the complications which are present or which follow and the age and resistance of the patient. The time which elapsed between the injury and the reduction of the fracture will also influence the prognosis. The prognosis should take into account several points: First, the effect of injury in respect to a favorable or unfavorable termination of the case; second, its simple or complicated course; third, the influence of each complication; fourth, the time required for recovery; and, fifth, the result as to normal occlusion of the teeth and normal functions of the jaws. The younger the patient, the more favorable the prognosis, because in the young fractures unite more easily and promptly than in the adult. If the fracture has existed two or three weeks previous to its reduction, and if there is considerable movement of the fragments, consolidation will not take place as rapidly after the parts have been brought into normal relation as it would if reduction were made at the time of the injury.

The presence or absence of infection plays a very important role in the duration of the treatment. In a simple fracture of the mandible where the fragments remain or are replaced in perfect contact, repair will take place at once without any untoward symptoms, without deformity or malocclusion, and without detriment to the functions of the jaws. If the fragments are not quite in apposition in simple fracture, but nearly so, the prognosis will be almost as favorable, although it will take somewhat longer for repair to take place and there may be some slight deformity manifested in malocclusion of the teeth.

All compound, comminuted and complicated fractures, which in their very nature present additional obstacles in the way of complete reduction, may not present so favorable a prognosis. In fractures of the superior maxilla, the fragments will not be subjected to muscle strain as those in the lower jaw, and the retention of the fragments in normal position will not be so complicated. Fractures of the condyle with displacement offer less in the way of favorable prognosis than any other fracture of the lower jaw. In these injuries there usually will be produced a traumatism in the temporomandibular joint which may later result in ankylosis.

Gunshot fractures of the jaws, which are necessarily in most cases compound and comminuted, are in a much less degree amenable to treatment than most other fractures. Splints for supporting the fragments must necessarily be more complicated, and infection is always present. All of these factors must be taken into consideration by the operator in forming a judicious prognosis in the treatment of fractures of the jaws.

Time will not permit us to discuss at length the treatment of fractures of the jaws. The treatment of these fractures consists in the fulfilment of three principal indications:

1. Reduce the broken fragments.
2. Retain the parts in normal relation until consolidation has taken place.
3. Prevent or control inflammatory processes.

No hard and fast lines can be drawn relative to the treatment of fractures of the jaws, for each case will present an entirely different problem.

In the treatment, the operator should aim to establish normal forms and normal function. The different forces, such as muscular tension, the force of gravity, movements of the tongue, etc., which tend to displace the fragments, must be taken into consideration in determining the methods of treatment.

Individual ingenuity must ever play an important role in the treatment of fractures of the jaws. Equally successful results will be obtained by means and methods that are wholly different. In the construction of splints, the aim should be so to design them that all parts of the mouth may be kept clean, without which normal repair will not take place.

